# Loneliness and the Associating Factors Among Regular Patients in Rural Community Hospitals: A Cross-Sectional Study

**DOI:** 10.7759/cureus.77723

**Published:** 2025-01-20

**Authors:** Ryuichi Ohta, Chiaki Sano

**Affiliations:** 1 Community Care, Unnan City Hospital, Unnan, JPN; 2 Community Medicine Management, Shimane University Faculty of Medicine, Izumo, JPN

**Keywords:** agricultural activities, bmi, community participation, depression prevention, japan, loneliness, primary care, rural health, social behaviors

## Abstract

Background

Loneliness is a significant precursor to depression and other adverse health outcomes, particularly in rural primary care settings. This study explores the association between loneliness and biopsychosocial factors among regular patients in rural community hospitals, focusing on Unnan City, Japan.

Methods

A cross-sectional study was conducted among 647 patients over 40 who regularly visited the general medicine department of Unnan City Hospital between September 1, 2023, and November 31, 2023. Data were collected using the Japanese version of the three-item University of California, Los Angeles (UCLA) Loneliness Scale and self-reported questionnaires on social behaviors, agricultural activities, community participation, eating habits, and physical health metrics. Logistic regression models identified factors associated with higher loneliness.

Results

Higher loneliness was significantly associated with frequent community dialogue (OR=2.43; 95% CI: 1.70-3.46; p<0.01) and frequent agricultural activities (OR=1.92; 95% CI: 1.27-2.90; p<0.01). In contrast, regular participation in community activities (OR=0.55; 95% CI: 0.39-0.79; p<0.01) was associated with lower loneliness. Lower BMI was also associated with higher loneliness (OR=0.95; 95% CI: 0.91-0.99; p=0.039).

Conclusion

Both social behaviors and physical health factors influence loneliness among rural patients. Effective interventions should emphasize the frequency and quality of social interactions, promote meaningful community participation, and consider physical health indicators like BMI. These findings can inform targeted strategies to reduce loneliness and prevent depression in rural healthcare settings.

## Introduction

Depression stands as a formidable challenge within primary care, casting a long shadow over the lives it touches [1]. Its insidious nature often makes it difficult to address effectively without understanding and intervening in its precursors [2]. Among these precursors, loneliness emerges as a critical condition, meriting attention for its profound impact on individual well-being and its potential to spiral into depression [3]. The link between loneliness and various adverse health outcomes, such as cardiovascular diseases and depression, underscores the necessity of managing loneliness within primary care settings as a preventative strategy [4]. By identifying and addressing the factors that contribute to loneliness, healthcare providers can take a proactive stance against the development of depression, enhancing patient care and improving overall health outcomes.

This research delves into the intricate relationship between loneliness and depression, with a specific focus on the primary care context. Loneliness, a condition marked by a significant discrepancy between desired and actual social connections, has been identified as a potent risk factor for depression [3,4]. The exploration of this relationship is particularly pertinent in primary care, where early detection and intervention can halt the progression of depression [5,6]. 

The relationship between loneliness and depression is complex and influenced by various factors. In primary care, several variables contribute to heightened loneliness, ranging from socioeconomic conditions to lacking engagement in communal activities [7-9]. Rural areas, in particular, present unique challenges due to their distinct social and economic landscapes, which can exacerbate feelings of isolation and loneliness [10]. Understanding these associated factors is crucial for developing targeted interventions that can mitigate loneliness and, by extension, prevent depression. However, the diversity of these factors and their variable impact in different contexts pose significant challenges to identifying and implementing effective interventions [11,12]. 

This study builds upon our previous work published in Cureus [8]. While both studies focus on the association between loneliness and social factors in rural settings, the current research differs in its focus, methodology, and analysis. Specifically, this study examines a broader range of biopsychosocial factors, including BMI. It emphasizes community dialogue, agricultural activity, and community participation, whereas the prior study centered primarily on community participation and its association with loneliness.

## Materials and methods

Study design

This cross-sectional study was performed with rural citizens who regularly visited a rural Japanese community hospital to clarify the association between feelings of higher loneliness and associated factors related to patients' usual lives.

Setting

Unnan City, situated in the southeastern region of Shimane Prefecture, Japan, is among the country's most rural areas. As of 2020, its population totaled 37,638, comprising 18,145 men and 19,492 women, with individuals aged over 65 accounting for 39% of the total. Projections indicate that this older population segment will rise to 50% by 2025. Unnan City has 16 clinics, 12 home care stations, three visiting nurse stations, and a public hospital, Unnan City Hospital. During the period of this study, the hospital operated 281 beds, including 160 for acute care, 43 for comprehensive care, 30 for rehabilitation, and 48 for chronic care [13]. Care services in Unnan City include 16 clinics, three visiting nurse stations, and 12 home care stations. Care managers work independently or with home care stations to coordinate care for home patients, collaborating with families and other professionals to assess care needs. Home care workers affiliated with these stations offer services such as physical care, assisted living, and transportation support [14].

Participants

This study included all patients aged over 40 who regularly visited the Department of General Medicine at Unnan City Hospital between September 1, 2023, and November 31, 2023 [8]. Participants included in this study were individuals aged 40 years and older who regularly attended the general medicine department at Unnan City Hospital. "Regular attendance" was defined as visiting the hospital thrice yearly for chronic disease management or annual health checkups. These criteria ensured participants were familiar with the hospital's services and had consistent healthcare access. This was relevant to the study's focus on biopsychosocial factors and loneliness in rural healthcare. To maintain the integrity of the data collected, we excluded patients who could not complete the study questionnaire independently. Specifically, individuals with documented severe cognitive impairment or dementia, as indicated in their medical records, were excluded due to potential challenges in reliably self-reporting their experiences.

Patients who declined to participate after receiving the study information or submitted incomplete questionnaires were excluded from the final analysis. These criteria ensured that the data accurately reflected the experiences of the study population. Data were collected from electronic medical records of patients visiting for chronic disease management or annual health checks. To assess loneliness and community activity participation, participants were provided with the Japanese version of the three-item University of California, Los Angeles (UCLA) Loneliness Scale questionnaire [15].

Participant selection

Between September 1, 2023, and November 31, 2023, 1024 patients were regularly followed by the general medicine department. The questionnaires were sent to all the patients. In total, 647 participants who answered the questionnaires were included in this study [8]. 

Data collection

Primary Outcome

The level of loneliness was measured using the Japanese version of the three-item UCLA Loneliness Scale, designed for community-dwelling adults (score range: 3-9). This scale includes three items: 1) How often do you feel you lack companionship?; 2) How often do you feel left out?; and 3) How often do you feel isolated from others? Each item is rated on a scale from one to three. The total loneliness score was determined by summing the scores from each item [15]. The internal consistency of this instrument, as reported in the validation study, was measured using Cronbach's alpha and found to be 0.83, indicating a high level of reliability [15].

Independent Variables

Data on social behaviors were collected through a questionnaire. For community dialogue, participants were asked: "How frequently do you engage in conversations with friends and community members?" Response options included less than once weekly, two to three times weekly, four to five times weekly, and more than five times weekly [8,10].

Regarding participation in community activities, participants responded to whether they engaged in local events such as salons, festivals, educational and cultural activities, and exercise programs (yes or no).

For agricultural activities, participants were asked about the frequency of their engagement in agricultural tasks, including personal gardening and crop cultivation, with response options of less than once weekly, two to three times weekly, four to five times weekly, and more than five times weekly [8,10].

Participants were also questioned about their exercise habits, specifically if they engaged in regular physical activity at least three times weekly for a minimum of 10 minutes per session (yes or no).

Eating habits were assessed by asking how many days per week participants consumed at least two meals with three components: a staple food, a main dish, and a side dish. Response options included less than once weekly, two to three times weekly, four to five times weekly, and more than five times weekly [8,10].

Participants' sleep habits were assessed by asking whether they made an effort to obtain sufficient sleep, which was defined as at least six hours per night (yes or no).

Background data were extracted from the electronic medical records of Unnan City Hospital, including age, sex, body mass index (BMI), serum creatinine level, estimated glomerular filtration rate (eGFR), and Charlson Comorbidity Index (CCI) for the severity of comorbidities [16]. The most recent laboratory data were used based on the participants' chronic disease follow-up or annual health checks [8].

Statistical analysis

Student's t-test was employed for analyzing parametric data, while the Mann-Whitney U test was applied for nonparametric data. Loneliness scores were dichotomized using the median value as the cutoff: ≥4 (higher loneliness) and <4 (lower loneliness), as the mean and median were similar (mean: 4.17; SD: 1.42; median: 4; interquartile range: 2). Community dialogue, agricultural activities, and healthy eating variables were also dichotomized: higher community dialogue (four or more times weekly) and lower community dialogue (less than four times weekly); higher agricultural activities (four or more times weekly) and lower agricultural activities (less than four times weekly); and higher healthy eating (four or more times weekly) and lower healthy eating (less than four times weekly). Univariate regression was used to assess associations between loneliness and independent variables, followed by a multivariate logistic regression analysis to explore the link between community dialogue frequency and loneliness. Variables with p-values <0.1 in univariate analysis were included in the multivariate model. The sample size was determined based on the total number of eligible patients who met the inclusion criteria during the study period (September 1, 2023, to November 31, 2023). Initially, 1024 patients were identified, and questionnaires were distributed to all. Of these, 647 patients completed and returned valid responses, resulting in a response rate of 63%. This number was deemed sufficient for the planned statistical analyses, including logistic regression, as it provided adequate power to identify meaningful associations between loneliness and biopsychosocial factors. The study design aimed to capture a comprehensive understanding of loneliness within a rural healthcare setting, and the sample size reflects the population available and willing to participate. Participants with any missing data were excluded, and statistical significance was set at p<0.05. All analyses were conducted using EZR (Saitama Medical Center, Jichi Medical University, Saitama, Japan), a graphical user interface for R (The R Foundation, Vienna, Austria) [17].

Ethical considerations

The hospital was assured of the anonymity and confidentiality of the patient information used in this study. Information related to this study was posted on the hospital website without disclosing any patient details. The contact information of the hospital representative was also listed on the website to ensure that any questions regarding this study were addressed. All participants were informed of the purpose of this study and provided informed consent. The Unnan City Hospital Clinical Ethics Committee approved the study protocol (approval code: 20230010).

## Results

Demographics of the participants

The group reporting higher loneliness included 337 individuals, while those reporting lower levels comprised 310 participants. A detailed analysis of demographic data revealed no significant age difference between these groups, with the mean age being slightly lower in the higher loneliness group (70.71 ± 12.87 years) compared to the lower loneliness group (71.85 ± 11.37 years; p=0.232). Gender distribution also did not significantly differ, with males representing 44.8% and 47.9% of the higher and lower loneliness groups, respectively (p=0.477). Regarding physical health metrics, body weight and BMI differed notably between the groups. Participants in the higher loneliness category had a significantly lower mean body weight (56.99 ± 13.07 kg vs. 59.68 ± 12.35 kg; p=0.007) and BMI (22.62 ± 4.07 vs. 23.41 ± 3.47; p=0.009), suggesting a potential link between lower body mass and increased feelings of loneliness. Other health parameters, including albumin levels and eGFR, showed no significant differences, indicating that the groups' basic nutritional status and kidney function were comparable. Chronic conditions such as diabetes mellitus, hypertension, and other comorbidities measured by the Charlson Comorbidity Index were assessed and found not to correlate significantly with loneliness, emphasizing that loneliness is likely influenced more by psychosocial factors than by chronic physical health conditions alone (Table 1).

**Table 1:** The demographics of the participants were categorized based on the amount of loneliness BMI - body mass index; CCI - Charlson Comorbidity Index; CKD - chronic kidney disease; COPD - chronic obstructive pulmonary disease; eGFR - estimated glomerular filtration rate; MI - myocardial infarction; SD - standard deviation; DM - diabetes mellitus

Factor	Total	Higher loneliness	Lower loneliness	p-value	t-value/χ²
N	647	337	310		
Age, mean (SD)	71.26 (12.18)	70.71 (12.87)	71.85 (11.37)	0.232	-7.41
Male sex, n (%)	299 (46.3)	151 (44.8)	148 (47.9)	0.477	-22.3
Albumin, mean (SD)	4.10 (0.41)	4.10 (0.41)	4.09 (0.41)	0.946	-0.37
Hight, mean (SD)	158.63 (8.60)	158.21 (8.51)	159.08 (8.70)	0.196	-6.92
Body weight, mean (SD)	58.28 (12.79)	56.99 (13.07)	59.68 (12.35)	0.007	-20.73
BMI, mean (SD)	23.00 (3.81)	22.62 (4.07)	23.41 (3.47)	0.009	-5.58
eGFR, mean (SD)	63.93 (15.49)	64.01 (16.00)	63.84 (14.94)	0.892	0.7
Hemoglobin, mean (SD)	13.28 (1.44)	13.25 (1.43)	13.31 (1.45)	0.592	-1.34
Hemoglobin A1c, mean (SD)	5.77 (0.56)	5.79 (0.60)	5.76 (0.50)	0.508	-0.68
LDL, mean (SD)	106.26 (23.85)	104.65 (23.87)	108.00 (23.74)	0.074	-25.97
Companionship, mean (SD)	1.54 (0.63)	2.04 (0.49)	1.00 (0.00)	<0.001	16.84
Isolated, mean (SD)	1.30 (0.51)	1.57 (0.58)	1.00 (0.00)	<0.001	9.67
Left over, mean (SD)	1.33 (0.52)	1.63 (0.57)	1.00 (0.00)	<0.001	9.03
Community participation, n (%)	211 (32.6)	82 (24.3)	129 (41.6)	<0.001	-123.58
Agriculture, n (%)					
Less than one time weekly	76 (11.7)	29 (8.6)	47 (15.2)	<0.001	-44.53
Two to three times weekly	70 (10.8)	24 (7.1)	46 (14.8)		
Four to five times weekly	169 (26.1)	89 (26.4)	80 (25.8)		
Every day	332 (51.3)	195 (57.9)	137 (44.2)		
Community dialogue, n (%)					
Less than one time weekly	109 (16.8)	34 (10.1)	75 (24.2)	<0.001	-108.22
Two to three times weekly	105 (16.2)	41 (12.2)	64 (20.6)		
Four to five times weekly	237 (36.6)	133 (39.5)	104 (33.5)		
Every day	196 (30.3)	129 (38.3)	67 (21.6)		
Healthy eating, n (%)					
Less than one time weekly	361 (55.8)	163 (48.4)	198 (63.9)	<0.001	-114.58
Two to three times weekly	144 (22.3)	89 (26.4)	55 (17.7)		
Four to five times weekly	116 (17.9)	64 (19.0)	52 (16.8)		
Every day	26 (4.0)	21 (6.2)	5 (1.6)		
Exercise, n (%)	267 (41.3)	141 (41.8)	126 (40.6)	0.811	6.82
Good sleep, n (%)	538 (83.2)	272 (80.7)	266 (85.8)	0.093	-37.78
CCI ≥ 5, n (%)	218 (33.7)	106 (31.5)	112 (36.1)	0.213	-35.24
CCI, n (%)					
0	20 (3.1)	13 (3.9)	7 (2.3)		
1	57 (8.8)	32 (9.5)	25 (8.1)		
2	82 (12.7)	44 (13.1)	38 (12.3)		
3	142 (21.9)	70 (20.8)	72 (23.2)		
4	128 (19.8)	72 (21.4)	56 (18.1)		
5	107 (16.5)	49 (14.5)	58 (18.7)		
6	64 (9.9)	30 (8.9)	34 (11.0)		
7	34 (5.3)	17 (5.0)	17 (5.5)		
8	10 (1.5)	8 (2.4)	2 (0.6)		
9	3 (0.5)	2 (0.6)	1 (0.3)		
Heart failure, n (%)	55 (8.5)	32 (9.5)	23 (7.4)	0.398	13.83
MI, n (%)	5 (0.8)	1 (0.3)	4 (1.3)	0.2	-5.6
Asthma, n (%)	43 (6.6)	28 (8.3)	15 (4.8)	0.084	25
Peptic ulcer, n (%)	55 (8.5)	27 (8.0)	28 (9.0)	0.674	-7.01
Kidney disease, n (%)	168 (26.0)	88 (26.1)	80 (25.8)	1	3.27
Liver disease, n (%)	52 (8.0)	26 (7.7)	26 (8.4)	0.774	-4.19
COPD, n (%)	38 (5.9)	17 (5.0)	21 (6.8)	0.404	-12.88
DM, n (%)	130 (20.1)	70 (20.8)	60 (19.4)	0.695	8.81
Brain infarction, n (%)	51 (7.9)	28 (8.3)	23 (7.4)	0.771	6.67
Brain hemorrhage, n (%)	13 (2.0)	5 (1.5)	8 (2.6)	0.405	-7.49
Connective tissue disease, n (%)	85 (13.1)	43 (12.8)	42 (13.5)	0.816	-3.7
Dementia, n (%)	12 (1.9)	8 (2.4)	4 (1.3)	0.388	6.41
Cancer, n (%)	69 (10.7)	37 (11.0)	32 (10.4)	0.899	4.41
Hypertension, n (%)	428 (66.2)	212 (62.9)	216 (69.7)	0.081	-50
Dyslipidemia, n (%)	388 (60.0)	196 (58.2)	192 (61.9)	0.336	-26.01

The result of the logistic regression analysis

The logistic regression analysis focused on identifying which factors were statistically significant predictors of higher loneliness among participants. The frequency of community dialogue emerged as a significant factor, with those engaging in dialogue four or more times weekly having higher odds of reporting loneliness (odds ratio (OR)=2.43; 95% confidence interval (CI): 1.70-3.46; p<0.01). In contrast, regular participation in community activities such as salons, festivals, and other social gatherings was associated with a reduced likelihood of feeling lonely (OR=0.55; 95% CI: 0.39-0.79; p<0.01). Participants who frequently engaged in agricultural activities reported higher levels of loneliness (OR=1.92; 95% CI: 1.27-2.90; p<0.01). Neither healthy eating habits nor regular exercise significantly impacted loneliness levels, indicating that these aspects of physical health are not directly associated with loneliness in this population. Furthermore, a lower BMI was associated with slightly reduced odds of loneliness (OR=0.95; p=0.039), underscoring the complex interactions between physical health and psychosocial well-being (Table 2).

**Table 2:** The multivariate logistic regression model with higher loneliness and associated factors BMI - body mass index; CCI - Charlson Comorbidity Index; COPD - chronic obstructive pulmonary diseases; CI - confidential interval

Factor	Odds ratio	95%CI	p-value
Frequent community dialogue	2.43	1.70-3.46	<0.01
Frequent community participation	0.55	0.39-0.79	<0.01
Frequent agricultural activity	1.92	1.27-2.90	<0.01
Healthy eating	1.18	0.78-1.77	0.44
Adequate sleep	0.80	0.51-1.25	0.32
age≧75	1.17	0.82-1.66	0.39
BMI	0.95	0.91-0.99	0.039
Asthma	1.62	0.82-3.23	0.17
Hypertension	0.85	0.60-1.21	0.37

## Discussion

This study investigated the multifaceted relationship between loneliness and several biopsychosocial factors among the rural population in Unnan City, Japan. Our findings highlight the complexity of social interactions and their impact on feelings of loneliness. Notably, frequent community dialogue was associated with increased feelings of loneliness. This finding highlights that the frequency of verbal interactions alone does not necessarily alleviate loneliness and may even exacerbate it when the interactions lack emotional depth or meaningful connection. Frequent but superficial conversations can remind individuals of unmet emotional needs or create a sense of isolation within social spaces. This aligns with existing research, which emphasizes that the quality and emotional support within interactions are critical for mitigating loneliness [18-20].

Conversely, active participation in community events, such as salons and festivals, was associated with a reduced likelihood of loneliness. These activities often provide structured opportunities for meaningful engagement, fostering a sense of belonging and shared experiences. Unlike frequent verbal interactions, which may lack emotional support, participation in social activities tends to involve deeper connections and shared purposes that are protective against loneliness. This finding underscores the importance of promoting quality social interactions that create a sense of community and fulfillment [21,22]. In rural contexts, people tended to respect community activities to realize their presence in their regions and be proud of their activities [23]. The advance of societies and modernization may reduce such trends in rural areas [24]. However, this study's results show the importance of community activities and collecting people in one activity to solve loneliness. This finding supports theories emphasizing the importance of engaging in socially and emotionally fulfilling activities to improve mental health.

The study also found a significant association between increased loneliness and frequent engagement in agricultural activities. Given the solitary nature of these activities in rural settings, this outcome could reflect the social isolation often accompanying such work [25]. This is consistent with literature indicating that while rural communities are usually close-knit, the physical and professional isolation associated with agricultural tasks can lead to significant feelings of loneliness [26]. Another research shows that not-frequent agricultural activities can mitigate loneliness conditions in rural settings [27]. Generally speaking, agricultural activities can promote their health conditions, so agricultural activities should be promoted based on each person's social conditions.

Neither regular physical activity nor dietary habits were significantly associated with loneliness. This suggests that while these factors are crucial for physical health, their impact on loneliness may be indirect and moderated by other variables such as social opportunities and community integration [6]. In addition, rural people can be healthier than in urban contexts because there are more natural resources, such as places to exercise and more nutritious food, than in urban areas [28]. This study's participants were patients in rural hospitals so they could be educated to exercise and have healthy eating habits.

An unexpected finding was the association between lower BMI and increased loneliness. This could suggest a bi-directional relationship where psychological distress impacts physical health and vice versa. This aligns with research suggesting that psychological stress can influence body weight and overall physical health [7,8]. This study's participants' mean BMI is 23 and within the standard range of healthy people, so lower BMI can show relatively thinner compared to general populations. Thinner bodies can be desired among young generations, but in the middle to older generations, they may give unhealthy and harmful images to others [29]. Such images may impinge on thinner people's social interaction with others, causing social isolation. Conversely, people with high loneliness may become unhealthy and thinner than others. Thus, regarding BMI and loneliness, the relationship should be investigated in longitudinal studies, and interventions should be adjusted to improve conditions with high loneliness, fitting each individual. 

Taken together, these findings reveal the multifaceted nature of social interactions in relation to loneliness. In the cultural context of rural Japan, frequent verbal interactions may be driven by social norms emphasizing harmony and community involvement but may lack the emotional depth needed to alleviate loneliness. Conversely, participation in structured social activities aligns with traditional rural practices that prioritize collective engagement and foster meaningful connections. These cultural dynamics underscore the importance of tailoring interventions to local traditions and social expectations to effectively address loneliness in rural populations.

This study has several limitations that must be acknowledged. First, the cross-sectional design limits our ability to infer causality. Longitudinal studies would be required to determine the directionality of the relationships observed. Second, the study relies on self-reported data, which may be subject to bias, including memory bias and social desirability bias. Third, the sample is limited to a rural Japanese population, which may limit the generalizability of the findings to other cultural or geographic contexts. Additionally, although validated, the measures used to assess loneliness and social interactions do not capture all nuances of social behavior and subjective feelings of isolation. Future research should consider using more comprehensive tools that can provide deeper insights into the quality of social interactions.

## Conclusions

This study highlights the complexity of factors influencing loneliness in a rural Japanese setting. Effective interventions to combat loneliness should increase the frequency and quality of social interactions, promoting meaningful and fulfilling social engagements. The association between physical health indicators like BMI and loneliness suggests the need for integrated health programs that address both mental and physical aspects. These findings contribute to a better understanding of loneliness dynamics in rural communities and underscore the need for targeted strategies that enhance both social and physical well-being.
